# Ultra-Fast and Optimized Method for the Preparation of Rodent Testicular Cells for Flow Cytometric Analysis

**DOI:** 10.1007/s12575-009-9003-2

**Published:** 2009-03-06

**Authors:** Rosana Rodríguez-Casuriaga, Adriana Geisinger, Beatriz López-Carro, Valentina Porro, Rodolfo Wettstein, Gustavo A Folle

**Affiliations:** 1 Departamento de Biología Molecular, Instituto de Investigaciones Biológicas Clemente Estable (IIBCE), CP 11600, Avda., 3318, Montevideo, Uruguay; 2 Facultad de Ciencias, Montevideo, Uruguay; 3 Servicio de Citometría de Flujo y Clasificación Celular (SECIF), IIBCE, Montevideo, Uruguay; 4 Unidad de Biología Celular, Instituto Pasteur de Montevideo (IPMONT), Montevideo, Uruguay

**Keywords:** Flow cytometry, Gene expression, Spermatogenesis

## Abstract

Homogeneity of cell populations is a prerequisite for the analysis of biochemical and molecular events during male gamete differentiation. Given the complex organization of the mammalian testicular tissue, various methods have been used to obtain enriched or purified cell populations, including flow cell sorting. Current protocols are usually time-consuming and may imply loss of short-lived RNAs, which is undesirable for expression profiling. We describe an optimized method to speed up the preparation of suitable testicular cell suspensions for cytometric analysis of different spermatogenic stages from rodents. The procedure takes only 15 min including testis dissection, tissue cutting, and processing through the Medimachine System (Becton Dickinson). This method could be a substitute for the more tedious and time-consuming cell preparation techniques currently in use.

## 1. Introduction

Spermatogenesis is a complex differentiation process essential for all the species with sexual reproduction, which leads to the formation of male gametes. In spite of its importance, a deeper comprehension of the molecular bases is still required for the understanding of the fundamentals of normal sexual reproduction, as well as for the treatment of testicular pathologies.

Spermatogenesis can be divided into three phases: mitotic proliferation of spermatogonia, meiotic divisions of spermatocytes, and spermiogenesis. Meiosis is a successful evolutionary widespread mechanism present in all eukaryotic species from fungi to superior plants and mammals. In sexually reproducing organisms, meiosis mediates the reduction in the DNA content of gametes, therefore compensating for doubling at fertilization [1].

The process of meiosis is mainly related to the behavior of chromosomes during prophase and anaphase of the first meiotic division, with highly significant events taking place (i.e., homologous synapsis, recombination, and segregation). Particularly, the homologous recombination that occurs during meiosis (crossing over) has vital importance as a means of genetic variability [2] and therefore constitutes a main source of biodiversity.

Different spermatogenic cell types with C (round spermatids, elongating and elongated spermatids, sperm), 2C (G1 spermatogonia, secondary spermatocytes), and 4C (different stages of primary spermatocytes, G2 spermatogonia) DNA content, coexist with somatic testicular cells (e.g., Leydig and Sertoli cells) in the testes of adult mammals. Cellular heterogeneity represents one of the major problems concerning the study of the molecular basis of mammalian spermatogenesis [3], together with the lack of spermatogenic cell lines for in vitro culture.

A strategy used to allow the analysis of gene expression along spermatogenesis has been the use of whole testes of prepubertal specimens, naturally enriched in early spermatogenic stages [4,5]. However, this approach does not precisely allow the assignment of specific transcripts to individual cell types. A sophisticated approach uses the differential light absorption pattern of the seminiferous tubule to isolate specific differentiation stages under a dissection microscope [6]. Although this approach has pioneered cellular and molecular analyses, it requires high expertise and does not separate the diverse cell types comprising each differentiation stage.

A different strategy has been the enrichment of cellular populations by Staput [7,8] or elutriation [3]. More recently, the identification and sorting of different spermatogenic cell subpopulations by flow cytometry have been described [9-12]. Cell separation techniques precisely allow studying which transcripts are present in a certain cell type, but have the disadvantage of involving laborious cell preparation procedures, and therefore, in some cases, expression levels may change to a certain extent during the purification process. Moreover, the duration of the process is especially critical for the representation of some RNAs and proteins with short half lives (reviewed in [13]).

The first step for any cell separation method is the preparation of a cellular suspension. The main goal of a cell suspension method is to provide a rich and representative sample of the different cellular subpopulations. Moreover, it is also important to maximize the number of viable cells and to prevent cell clumps. In the case of testicular cell suspensions, it is critical to avoid selective damage of specific cell types and to minimize the formation of multinucleate cells [3] which tend to form because of the syncitial nature of the seminiferous epithelium. Besides, when the cell suspensions are prepared for downstream applications such as gene expression studies, the duration of the process and the handling involved must be taken into consideration, in order to prevent degradation or loss of macromolecules of interest.

Various protocols using mechanical dissociation for the preparation of testicular cell suspensions have been described [7]. However, suspensions prepared by these methods tend to aggregate very quickly, and the yield of viable cells is at most 80% (generally less). Even more important, certain cell types may be selectively damaged [3]. Additionally, since manual mechanical methods are operator-dependent, results are not highly reproducible. Methods involving a combination of gentle mechanical action with enzymatic treatment have rendered better results in terms of yield and cell type representation, while minimizing cell aggregation [3,9]. Nevertheless, they are time-consuming and involve a lot of handling.

Here, we present a very simple and efficient protocol to rapidly obtain a cellular suspension from testis material for flow cytometric analysis. This protocol eliminates steps between animal sacrifice and spermatogenic stage-specific molecular studies, aiming to optimize macromolecule preservation.

## 2. Materials and Methods

### 2.1. Animals

Male CD1-Swiss outbred stock mice (7–9 weeks old), Sprague–Dawley rats (8–10 weeks old), and Dunkin Hartley guinea pigs (12–14 weeks old) were used as a source of normal adult testes for all the experiments. Testis material from one specimen was used for each experiment. Between seven and nine adult individuals of each species were analyzed while two 21-day-old rat pups were processed for immature testis studies. Animals were sacrificed by cervical dislocation (rats and mice) or by administration of an overdose of sodium pentobarbital (guinea pigs), following the recommendations of the Uruguayan National Commission of Animal Experimentation (CHEA).

### 2.2. Preparation of Cellular Suspensions

Testes were dissected into 96 mm glass Petri dishes containing ice-cold separation medium [10% fetal calf serum in Dulbecco's Modified Eagle's medium, containing high glucose and L-glutamine], and cut into 2–3 mm^3^ pieces after removal of the tunica albuginea. Four to five of these pieces were immediately placed into a disposable disaggregator Medicon™ with 50 μm separator mesh (Becton Dickinson) plus 1 mL of ice-cold separation medium and processed for 50 s in the Medimachine System (Becton Dickinson). Each Medicon unit contains a fixed stainless-steel screen with about 100 hexagonal holes surrounded by six microblades. The tissue is brought to each hole by a metal rotor inside the Medicon chamber and disaggregated by passing over the sharpened holes and through the metal screen, while a micropump under the screen supplies liquid and flushes out the holes.

The cell suspension was recovered from the Medicon unit with a 5-mL disposable syringe, subsequently filtered through a 50-μm Filcon (Becton Dickinson) and 25 μm nylon mesh, and placed on ice. Cells were counted by means of a Neubauer chamber and diluted to a concentration of 1–2 × 10^7^ cells/mL in separation medium. 2-Naphthol-6,8-disulfonic acid, dipotassium salt (NDA; Chemos GmBH, Regenstauf, Germany) was added to the suspension to a final concentration of 0.2% in order to prevent cell clumping. Cell viability of the testicular cell suspensions was measured with the trypan blue dye exclusion test and the LIVE/DEAD viability kit for animal cells (Molecular Probes, Eugene, OR, USA) according to manufacturer's instructions.

### 2.3. Flow Cytometric Analysis

Prior to flow cytometric analysis, the vital dye Hoechst 33342 (Sigma-Aldrich, St. Louis, MO, USA) was added to cell suspensions to a final concentration of 5 μg/mL and incubated for 10 min at 37°C in the dark. Cells were analyzed by means of a MoFlo Cytometer (DakoCytomation) equipped with a UV excitation wavelength laser (Innova 90C-6) operating at 25 mW and a 70-μm nozzle.

Analysis of the following parameters was performed with Summit v4.3 software: forward scatter (FSC-H); side scatter (SSC-H); pulse-area or total emitted fluorescence (FL2-A); and pulse-high or intensity of fluorescence emission (FL2-H). Instrument linearity and doublet discrimination performance was checked with DNA QC particles (Becton Dickinson) stained with Hoechst 33342.

### 2.4. Preparation of Samples for Microscopy

Cell morphology of unstained fresh material was analyzed by phase contrast microscopy with an Olympus IX81 motorized inverted research microscope equipped with a ×40/0.4 UIS2 Plan acromat objective and an Orca AG camera (Hamatsu Photonics C4742–80–12A6). Images were captured using Image ProPlus v.6.0 software.

## 3. Results and Discussion

Table 1 shows a comparison between the method presented here for the preparation of testicular cell suspensions and previously described ones [e.g., [7,9,14]]. Briefly, the main advantages of our protocol are:

**Table 1:** Comparison of different methods for the preparation of testicular cell suspensions

	Lam et al. 1970	Meistrich 1972	Malkov et al. 1998	This work
Duration	FAST (~30 min)	Time-consuming (~1 h)	Time-consuming (~2 h)	Very fast (~15 min)
Handling	Significant	High	High	Minimal
Reproducibility	Variable	Variable	Variable	Very high
Cell debris	Significant	Moderate	Not shown	Scarce
Multinucleates	Significant	Significant	Significant	Scarce
Viability	≤80%	98%	Not determined	>85%
RNAses	No	No	Yes	No
Trypsin/collagenase	No	Yes	Yes	No

**Figure 1:**
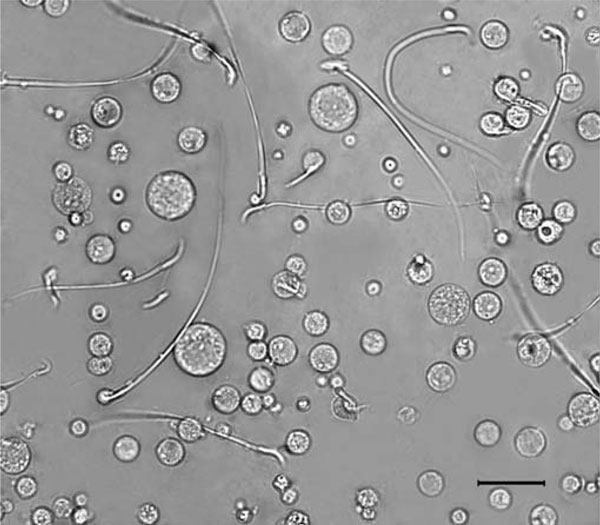
**Partial view of a cell suspension from adult rat testis**. The suspension was prepared with the Medimachine as described in the "Materials and methods" section and visualized by phase contrast microscopy. The wide variety of cell sizes and shapes can be observed in a well-dissagregated state. As can be seen, most of the spermatozoa keep their flagellae. The absence of multinucleates and the integrity of cell cytoplasms are also evident. The *bar* corresponds to 25 μm.

**Figure 2:**
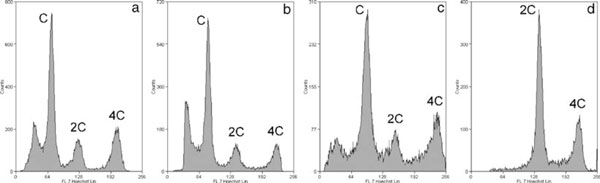
**Flow cytometric DNA content analysis of rodent testicular cell suspensions**. Cell suspensions were prepared from adult rats (**a**), mice (**b**), guinea pigs (**c**), and from 21-day-old rat pups (**d**), and stained with the vital dye Hoechst 33342. Three main subpopulations of cells can be easily distinguished in the histograms obtained for adults of the three species (**a**, **b**, **c**), according to their DNA content (C, 2C, and 4C). The additional peak to the left of the C subpopulation corresponds to elongating spermatids and elongated condensed spermatozoa, as previously reported [9]. The immature nature of the 21-day-old rat testis (**d**) is evident, since the C subpopulation is absent.

1. Testicular cell suspensions prepared using the Medimachine as described in the "Materials and methods" section are obtained in only 15 min; this includes testis dissection, tissue cutting. and Medimachine processing. This time span represents approximately one-eighth of the time usually taken by other protocols [e.g., [9]]. The brevity of this protocol would account for the good preservation of short-life macromolecules (e.g., some mRNAs), which is critical when a representative sample of compounds present in the original cell population is required.

2. Since the method involves minimal handling of the material, it is easily reproducible and, again, would prevent RNA degradation during the process.

3. Unlike most currently used protocols for preparation of cellular suspensions from testis, the method presented here does not involve enzymatic action. The enzymes generally included are collagenase and mainly trypsin which, even though they contribute to an adequate disaggregation of the tissue [3], they can also affect cell integrity and/or preservation of macromolecules of interest. The lack of enzymatic treatments not only favors cell and protein preservation, but also makes the protocol cheaper.

4. Very little clumping and debris are observed in cellular suspensions. Although it has been previously reported that mechanically prepared cell suspensions clumped more readily than tripsinized ones [3], we have found that our protocol led to well-disaggregated cell suspensions in the absence of enzymatic action (Figure 1). Moreover, the inclusion of NDA [3] all along the process has proved to be very helpful, not only to prevent cell clumping but also to avoid nozzle clogging during flow studies. The good quality of cell suspensions was also evidenced by cytometric analysis, as judged by the minimal cell debris observed for the three examined species (Figure 2).

5. Very few multinucleates are also observed. According to Meistrich, about 13% of the round spermatids are found as multinucleates either when cell suspensions are prepared by an exclusively mechanical method [14] or in the presence of trypsin [15]. Our observations indicate that cell suspensions obtained by the Medimachine method rendered very scarce multinucleates (see Figure 1). This is most probably due to reduced handling, since it has been stated that multinucleates are produced mainly as a consequence of tissue manipulation [3].

6. The use of a vital dye staining makes the populations suitable for subsequent sorting and RNA extraction. Although Hoechst 33342 concentrations ranging from 1–10 μg/mL and 20–90 min incubation times are usually recommended to obtain DNA histograms with acceptable coefficients of variation (CV), good results were achieved analyzing testicular cell populations with 5 μg/mL dye concentration and reduced incubation time (10 min). The optimal dye concentration and staining time for different cell types vary as dye uptake depends on cellular metabolic rates. Hoechst 33342 noncovalent DNA minor groove binding requires an equilibrium between intracellular free-dye and DNA-bound dye for stoichiometric DNA binding in live cells [[16] and references therein]. Since a short incubation time was desirable to minimize cell death and damage to short-lived macromolecules, this minimal incubation time is considered an important advantage. An additional benefit is the A-T base pair preference of Hoechst 33342 which results in nearly no RNA-associated signal, and therefore no need for RNase treatment.

Even though the Medimachine mechanical protocol proved to be faster and easier than previously described methods, a comparative analysis concerning the quality of cell suspensions still had to be performed. For that purpose, we decided to assess the viability, integrity, and cell type proportions of the cellular suspensions obtained after Medimachine treatment.

The viability of the suspensions was above 85% when tested by means of the Live/Dead kit (Molecular Probes) and the trypan blue dye exclusion test. This was considered totally acceptable, since other mechanical methods used in the past rendered less or, in the best cases, similar viability levels [7,14]. Moreover, the Live/Dead kit's viability level—which is equivalent to the percentage of intact cells—evidences good cytoplasm preservation (also seen in Figure 1).

On the other hand, the histograms obtained by flow cytometry analysis of testicular cell suspensions from adult rats and mice prepared using the Medimachine mechanical method (Figure 2a, b) did not show significant differences with previously described gentle mechanical plus enzymatic protocols [9]. This supports the assumption that our method does not selectively damage any specific cell type. Furthermore, when the cellular composition of adult guinea pig testicular cell suspensions obtained through the Medimachine was analyzed by flow cytometry (Figure 2c), a coincidence was found with the information we have reported by cell counts in cross sections of seminiferous tubules embedded in Epon [17] (Table 2).

**Table 2:** Comparison of the relative percentages of cell subpopulations in adult guinea pig testes by two methodological approaches

**Counts on cross-sections of seminiferous cords **[17]	Flow cytometry (this work)
C = 66.5%	C = 65.5%
2C = 11.0%	2C = 11.5%
4C = 22.5%	4C = 23.0%

C, 2C, and 4C refer to DNA content

Moreover, when the method was applied to the analysis of the cellular composition of immature testes, the results were also in agreement with previous reports. As an example, the relative percentages obtained by our method for testicular subpopulations differing in DNA content (C, 2C, and 4C) for 21-day-old rats, were 0%, 65%, and 24%, respectively (Figure 2d). The corresponding previously reported values were 0%, 77%, and 19% for 20-day-old rats, and 0%, 48%, and 47% for 22-day-old rats [9]. Therefore, we have obtained intermediate relative percentages of C, 2C, and 4C cells for an intermediate developmental stage, which would again indicate that our method does not selectively damage any specific cell type.

Thus, the viability and cell type proportions in the cellular suspensions obtained with the mechanical method described here do not significantly differ from the previously reported results using more time-consuming and laborious approaches. Besides, the reproducibility of the method was very high, specially if individual variations are taken into account (Table 3).

**Table 3:** Reproducibility analysis of the method described here

	DNA content		
	
*Rattus norvegicus* specimen	C	2C	4C
1	72.0	12.0	16.0
2	76.0	11.0	13.0
3	75.0	13.0	12.0
4	76.0	11.0	13.0
5	72.0	12.0	16.0
6	71.5	15.0	13.5
7	73.0	13.0	14.0
Arithmetic mean	73.6	12.4	14.0
Standard deviation	2.0	1.4	1.5

Relative percentages of C, 2C, and 4C cell populations from seven independent experiments performed with adult rats (one individual per experiment) are shown as an example

Summarizing, we have developed a very simple, fast, and reproducible cell suspension-preparation method for flow cytometry analysis of rodent testicular cell populations. Although not tested here, this method could be also used in combination with other cell purification techniques such as elutriation or staput. Additionally, since this method involves very little manipulation and avoids enzymes and detergents, it could become an ideal choice for delicate downstream applications such as gene expression studies. In this regard, we are now using sorted spermatogenesis cell populations (see supplemental Figure 1, accessible at http://www.iibce.edu.uy/PAPERLINKS/RRCasuriaga-supplemfig1.jpg) for reverse transcriptase-polymerase chain reaction amplification of stage-specific transcripts (to be published elsewhere). Although we have not tested the preservation and/or specificity of any spermatogenic protein yet, our method could also be advantageous for protein studies considering the absence of trypsin in the procedure.

In conclusion, time savings, little manipulation involved, and the quality of the resulting suspension make this method an interesting alternative to the more tedious and time-consuming cell preparation techniques currently in use.

## Appendix

### Protocols

Preparation of cell suspensions

   Materials:

- Glass Petri dishes

- Scissors and forceps

- Medimachine (BD)

- Medicon units (BD)

- 50 μm nylon membrane or 50 μm Filcon units (BD)

- 25 μm nylon membrane or 25 μm Filcon units (BD)

- 5 mL syringes

- Neubauer chamber

- Separation medium: Dulbecco's Minimal Essential Medium (D-MEM ) supplemented with 10% fetal calf serum

- NDA (2-naphthol-6,8-disulfonic acid, dipotassium salt)

Procedure:

1. Place the dissected testis in a 96-mm glass Petri dish on ice, containing 10 mL of ice-cold separation medium.

2. Remove the tunica albuginea and cut the decapsulated testis into 2–3 mm^3^ pieces.

3. Place four to five of these pieces in a disposable disaggregator Medicon™ (BD) along with 1 mL of cold separation medium and process in the Medimachine system for 50 s.

4. Recover the resulting cell suspension using a 5-mL syringe without needle.

5. Filter through a 50-μm nylon mesh [or Filcon™ unit (BD) containing a similar mesh], previously soaked with 0.5 mL of separation medium.

6. Repeat step 5 but using a 25-μm nylon mesh (or equivalent BD Filcon™ unit).

7. Take an aliquot of the cell suspension to count in a Neubauer chamber and adjust cellular concentration to 1–2 × 10^7^ cells/mL.

8. Add NDA to a final concentration of 0.2% to avoid cell clumping.

### 1.2. Cell viability evaluation

Materials:

- LIVE/DEAD viability kit for animal cells (Molecular Probes, Eugene, OR, USA)

- Trypan blue

Procedure:

Check cell viability of the testicular cell suspensions with the LIVE/DEAD viability kit for animal cells (Molecular Probes, Eugene, OR, USA) following manufacturer's instructions. Alternatively, trypan blue dye exclusion test can be performed as detailed below:

1. Take 0.1 ml of the concentrated cell suspension and dilute it to an approximate concentration of 1-2 × 10^5^ cells/mL.

2. Add 0.1 ml of 0.4% trypan blue stain to 0.5 mL of the diluted suspension. Mix thoroughly.

3. Allow to stand 5 min at room temperature.

4. Fill a hemocytometer as for cell counting.

5. Under a microscope, count nonviable (stained) and viable (unstained) cells.

### 1.3. Flow cytometry analysis

Materials and equipment:

- Hoechst 33342 (Sigma-Aldrich, St. Louis, MO, USA), stock 5 mg/mL

- MoFlo Cytometer (DakoCytomation) equipped with a UV excitation wavelength laser (Innova 90C-6)

Procedure:

1. Prior to flow sorting, add Hoechst 33342 to the cell suspension to a final concentration of 5 μg/mL. Incubate for 10 min at 37°C in the dark.

2. Perform cell analysis by means of a MoFlo Cytometer (DakoCytomation) equipped with a UV excitation wavelength laser (Innova 90C-6) operating at 25 mW. Prior to cell analysis, check instrument linearity and doublet discrimination performance with DNA QC Particles (Becton Dickinson) stained with Hoechst 33342.

3. Use Summit v4.3 software (or a similar one) to analyze the following parameters: forward scatter (FSC-H); side scatter (SSC-H); pulse-area or total emitted fluorescence (FL2-A); and pulse-high or intensity of fluorescence emission (FL2-H).

## Supplementary Material

Supplemental Figure 1 **Partial view of sorted 4C guinea pig cells**. The cell suspension was stained with Hoechst 33342 (10 min) and analyzed with a MoFlo cytometer (DakoCytomation) equipped with a UV excitation wavelength laser (Innova 90C-6) operating at 25 mW and a 70-μm nozzle. Sorted cells were recovered onto 12 × 75 mm polystyrene tubes, centrifuged at 450 g (5 min) and 0.5 ml of paraformaldehyde (1%) were added to the cell pellet. Aliquots of fixed cells were dropped onto clean microscope slides and nuclei stained with Giemsa (3%, 5 min). Note the similarity of nuclei size and morphology (GIF 1573 kb) Click here for file
