# Supplementary figures and images for: Ultra-Fast and Optimized Method for the Preparation of Rodent Testicular Cells for Flow Cytometric Analysis
Source: Biol Proced Online. 2009 Mar 6;11:184–95. doi: 10.1007/s12575-009-9003-2 (PMC3055716; doi:10.1007/s12575-009-9003-2)

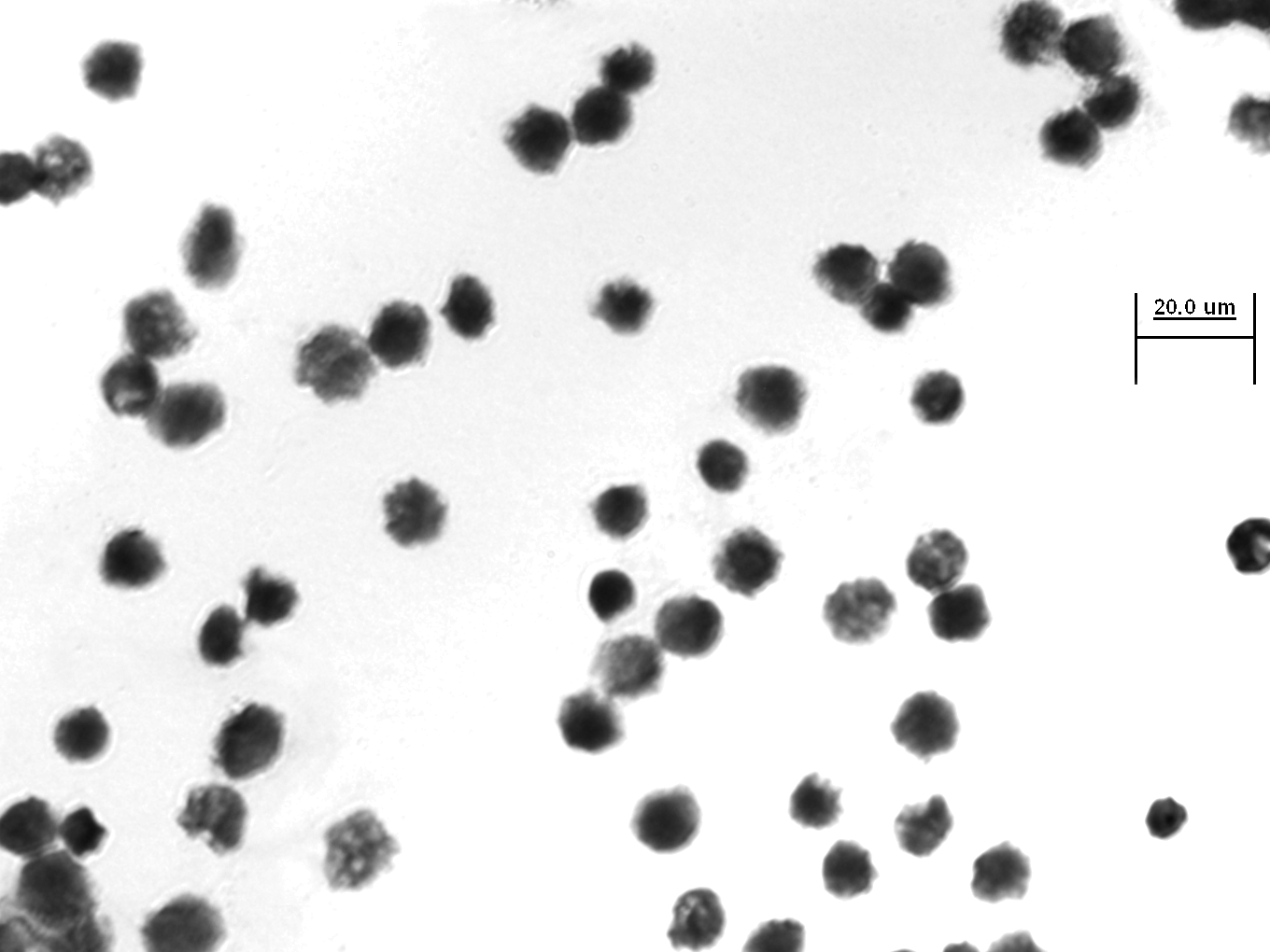

Supplement: Supplemental Figure 1 — Partial view of sorted 4C guinea pig cells. The cell suspension was stained with Hoechst 33342 (10 min) and analyzed with a MoFlo cytometer (DakoCytomation) equipped with a UV excitation wavelength laser (Innova 90C-6) operating at 25 mW and a 70-μm nozzle. Sorted cells were recovered onto 12 × 75 mm polystyrene tubes, centrifuged at 450 g (5 min) and 0.5 ml of paraformaldehyde (1%) were added to the cell pellet. Aliquots of fixed cells were dropped onto clean microscope slides and nuclei stained with Giemsa (3%, 5 min). Note the similarity of nuclei size and morphology (GIF 1573 kb) [file 1480-9222-11-1-9003-S1.tif]
